# Functional and Bioactive Properties of Food: The Challenges Ahead

**DOI:** 10.3390/foods7090139

**Published:** 2018-08-31

**Authors:** Diego A. Moreno, Nebojsa Ilic

**Affiliations:** 1 CEBAS-CSIC, Phytochemistry and Healthy Foods Lab, Department of Food Science & Technology, Campus Universitario Espinardo–25, E-30100 Espinardo, Murcia, Spain; 2 Institute of Food Technology, University of Novi Sad, Bul.cara Lazara 1, Novi Sad 21000, Serbia; nebojsa.ilic@fins.uns.ac.rs

With the idea and objective of bringing together new data on biomolecules from fruits, vegetables, wild or medicinal plants, and other organisms (either from land or marine origin) which can exert functional and health-promoting effects through bioactivity beyond the basic nutrient composition, we edited this special issue on “Functional and Bioactive Properties of Food” (URL: http://www.mdpi.com/journal/foods/special_issues/Functional_Bioactive_Properties_Food). The evidence presented in the participating papers is still not enough to demonstrate causality and functionality because the multifactorial conditions of the diseases (which are not related to a single effect or compound) are still a big challenge for this generation of scientists involved in the research of bioactives from foods for nutrition and health.

The principle of “safety first” clearly drives the research on bioactive compounds of natural origin to be incorporated in food and feed formulas for health and wellbeing. Besides bioaccessibility and the demonstration of biological activity, it is clear that an early positive result in any new development would clear up any safety or toxicology issues. One example is the use of ginger rhizome in early pregnancy [1]. More research is needed regarding its efficacy and safety, even though the available data suggests that ginger is a safe and effective treatment for nausea and vomiting in women during early pregnancy [1]. On the other hand, daily food consumption could lead to unexpected exposure of contaminants, such as heavy metals, and the use of plant-derived food formulas could help in the reduction of damage in the human body through different mechanisms of detoxification, as in the use of Selenium-enriched rice grass juice to overcome Cadmium (Cd) contamination in foods [2], even though the evidence is generated in vitro and further developments are expected in the future.

The use of highly-sensitive techniques to fully characterize the components in foods, formulations, and ingredients, as well as to determinate their bioaccessibility, bioavailability, metabolism, and biological activity, is another supporting structure of current and future challenges of the research and development of foods for health. In the separation of compounds in the complex mixture of any given food or formula, as well as in the evaluation of the different effects of growth conditions, agronomical practices, farming, production of any kind, and processing, either at the industrial or the domestic level, must be taken into consideration. The highly-sensitive separation conditions of the fatty acids in digested and processed milk [3], the attention given to the effects of processing on the content of bioactives as the betaine content in gluten-free grains and products [4] has possibilities of fortification, and the necessary phytochemical characterization of bioactive compounds in quite unknown and newly-used fruits of Pitanga [5], are only a few examples of the current trends in the search for bioactive-rich, non-animal-origin foods for health. Nowadays, where everything in terms of foods are connected or related to functionality, a clear, suitable, and effective set of techniques are needed to really highlight the real or potential functionality of a given product, and methods are continuously evolving in terms of accuracy and time consumption, from the preparation of samples to the elucidation of structures and mode of action. Once gathered, the information and data in the discussion should be relevant in terms of the significance of the results, not only at the statistical level but also regarding the physiological and biological relevance of the findings in terms of associative cause–effect between bioactive and bioavailable metabolites and the expected function in target organs. Besides the approaches in improving the scientific work to back-up the results, there is a need and clear evolution in the methodologies too in terms of respect to the environment and hazards in the working environment, with more and more conscious labs using greener alternatives to implement sustainable practices from the farm to the lab and the clinical environments. The design of “smart-foods” also involves nanotechnology to improve the bioavailability, metabolism, and bioactivity of foods, and food components specially designed to target tissues affected from malignancies where the safety and innocuousness of the encapsulated bioactives is the “stone in the road”, as seen in the review of Martínez-Ballesta et al. [6] and references therein.

The current trend of producing foods which are aimed at more efficient diets, reduction of intake of products from animal origin, focus on functionality, novel zero-waste processes, and the appreciation of the complex composition and structure of foods and ingredients to be investigated in the context of free-living people using dietary recommendations and dosages compatible with everyday living to treat, managing and modulating the complex conditions of non-communicable diseases from the perspective of prevention is somehow utopic at the present time. However, the aim and will of thousands of research teams from many different areas of science and technology are looking in that direction. Examples of “potential” functional components in foods are included in the special issue of Yamane et al. [7] regarding the probiotics in Kefir, or the healthy lipids in rice Koji extract [8], though the results are obtained through in vitro models—thus being very far from becoming a real functional food or ingredient in the short term. Based on the works related to experiences in humans about functionality, the use of spices in real life did not seem negative [9], but also far from functional. The search for the right delivery system or “attractive” matrix for the consumer, such as cookies enriched with green tea [10], may be acceptable from the organoleptic point of view—however, the biological relevance of the effects on glucose metabolism is scarcely significant. A systematic review on the use of alpha-amylase inhibitors from white bean for body weight and body fat [11] shows only limited reductions, and the limitations of human interventions need better designs and the development of sensible biomarkers to help in the understanding of the connections between foods, nutrition, and health.

In summary, the development of new ingredients and foods enriched with bioactive compounds, having health-promoting characteristics, offers new visions and opportunities for alternative strategies in food production, supply, and consumption. Following this development and the worldwide trend toward foods for nutrition and health, there is a need for a multidimensional and multidisciplinary approach when it comes to connecting foods and their functionality. Among them, of particular importance is performing more in vivo studies showing how functional ingredients and foods actually perform in a living organism, thus demonstrating its bioactivity and functionality. Connected with in vivo studies will be a growing need to perform metabolomic ones that will help to elucidate how these functional ingredients influence metabolism, as well as to explain the possible modes of action. In the end, as most authors stated, further research is required in all and every one of the presented papers in the “Functional and Bioactive Properties of Food”, and this assures an exciting time for the researchers in this field and for the general public interested in the relationship between food and health.
